# Music Enjoyment in Hearing Loss: Beyond Auditory Perception

**DOI:** 10.3390/brainsci16090945

**Published:** 2026-09-05

**Authors:** Meredith Kossoff, Kevin Bryan, Veronica Zhang, Emily Gao, Alexander Chern

**Affiliations:** 1 Department of Otorhinolaryngology–Head and Neck Surgery, Hospital of the University of Pennsylvania, Philadelphia, PA 19104, USA; meredith.kossoff@pennmedicine.upenn.edu (M.K.); kevin.bryan@pennmedicine.upenn.edu (K.B.); veronica.zhang@pennmedicine.upenn.edu (V.Z.); 2 Department of Otolaryngology–Head and Neck Surgery, Johns Hopkins University School of Medicine, Baltimore, MD 21205, USA; egao7@jh.edu

**Keywords:** hearing loss, music, music perception, music enjoyment, music reward, music psychology, patient-reported outcome measure, quality of life

## Abstract

**Highlights:**

**What are the main findings?**

- Music enjoyment is a multidimensional construct with a basis in hearing loss research, music psychology, and neuroscience.
- Hearing loss research on music enjoyment has emphasized auditory perception; however, a growing body of music psychology and hearing loss research demonstrates that music enjoyment and perception are related but diverging concepts.

**What are the implications of the main findings?**

- A broader music enjoyment definition is needed to advance future research, outcome measurements, and clinical care.
- Development of a patient-reported outcome measure for music enjoyment could enable assessment of music enjoyment across the hearing spectrum.

**Abstract:**

**Background/Objectives:** Music is an important component of quality of life and is increasingly recognized as a meaningful patient-centered outcome in hearing healthcare. Although individuals with hearing loss frequently report reduced enjoyment of music, research in this area has focused on auditory perception, including pitch, timbre, melody, and sound quality. Consequently, music enjoyment is often conceptualized and measured primarily through perceptual performance, despite growing evidence that enjoyment represents a broader subjective experience. **Methods:** This narrative review examines the current conceptualization of music enjoyment in individuals with hearing loss by integrating literature from audiology, music psychology, affective neuroscience, and patient-reported outcome measurement. **Results:** We discuss music enjoyment as a multidimensional construct influenced by, but not limited to, auditory perception. Additional determinants include emotional reward, musical engagement, prior musical experience, expectations, identity, listening behaviors, and social context. We further examine evidence demonstrating that music perception and music enjoyment, while related, may diverge substantially across individuals with hearing loss, highlighting the limitations of equating perceptual fidelity with patient experience. Finally, we consider the implications of this conceptual distinction for hearing rehabilitation and music-related outcome measurement. **Conclusions:** We argue that a broader patient-centered understanding of music enjoyment is necessary to guide future research, inform clinical practice, and support the development of next-generation patient-reported outcome measures capable of capturing the complexity of music experience in individuals with hearing loss.

## 1. Introduction

Hearing loss (HL) is a highly prevalent sensory disorder that is associated with social isolation, cognitive decline, and dementia [1,2,3,4,5,6,7,8]. Individuals with HL experience functional hearing difficulties that affect daily activities central to well-being [9]. Engaging with music is a universal and meaningful part of human life associated with improved quality of life and overall well-being [10,11]. For individuals with HL, music listening is often more challenging, but it remains an important and valued activity [1,12,13,14]. The HL population has decreased engagement in musical activities and poorer ratings of music enjoyment compared to individuals with normal hearing [12,13,15,16]. Despite these challenges, many patients view music as an important aspect of life and a meaningful component of successful hearing rehabilitation [13,14,17].

As hearing science has evolved, functional outcomes of hearing beyond speech perception, such as music-listening, have received increasing attention [18,19]. Research on music and HL has emphasized music perception (the ability to detect and interpret elements of music such as pitch, rhythm, melody, and timbre), particularly among specific subgroups of HL such as cochlear implant (CI) users [13,17,20,21,22,23,24]. As such, many assessment tools are designed to evaluate these domains [25,26]. These perceptual abilities are important, as music presents unique auditory demands that are particularly vulnerable to HL [27,28,29]. Yet, an emphasis on perception alone may not fully capture patients’ music listening experience and enjoyment [30,31,32].

Evidence suggests that music perception and music enjoyment are related but distinct constructs. Music enjoyment is a multidimensional construct, and individuals with similar perceptual performance may report markedly different levels of enjoyment [1,15,17,33,34,35]. Personal, emotional, behavioral, and social factors shape musical enjoyment in different ways than perceptual ability alone [1,23,24,33]. These findings suggest that a broader conceptualization may be necessary to understand music enjoyment in HL. The objectives of this narrative review are to: (1) synthesize multidisciplinary concepts of music enjoyment from hearing loss research, psychology, and neuroscience; (2) assess the unique challenges that music perception poses for individuals with HL; (3) present music enjoyment as a multidimensional construct that diverges from auditory perception; and (4) discuss implications of this broader conceptualization for hearing rehabilitation and the development of music-related outcome measures.

## 2. Materials and Methods

This narrative review examined the multidisciplinary literature regarding music perception and music enjoyment. A narrative approach was selected to synthesize and compare findings across hearing loss research, psychology, and neuroscience. In keeping with narrative review methodology, a comprehensive review of published literature was conducted [36,37]. Searches were performed in PubMed, Scopus, and Cochrane from inception through 20 July 2026. The following search terms were used alone and in combination: “music enjoyment,” “music perception,” “music reward”, “music appreciation,” “music appraisal,” “music psychology,” “neuroscience,” “hearing loss,” “pitch,” “timbre,” “temporal processing,” “hearing aid,” and “cochlear implant.” Search queries and initial number of results are included in Appendix A. Reference lists of included articles were screened to identify other additional potential articles. Eligible studies included English-language peer-reviewed articles that contributed to an understanding of music perception and enjoyment in the context of hearing, psychology, neuroscience, or patient-reported outcome measurement. Reviews, observational studies, and experimental studies were included. As a narrative review, there was no systematic method for study selection, and a formal risk-of-bias assessment was not performed. Studies were selected based on their relevance to the review objectives and topics. For neuroscientific and hearing topics, priority was given to studies which provided mechanistic evidence. For music psychology and music-related HL literature, priority was given to studies that directly informed the concepts of music enjoyment from a psychology perspective, music perception as compared to music enjoyment, and music-related outcome measurement. Contemporary review articles were used to identify major conceptual frameworks and themes, areas of consensus and ongoing investigation, and provide context for interpretation of primary studies. Information from included studies was used to inform the structure of this review and craft a narrative synthesis.

## 3. What Is Music Enjoyment?

Music enjoyment (also referred to as music reward or music appreciation) is a multifaceted, complex concept that does not have a universally recognized definition. At its core, music enjoyment involves the pleasurable, emotionally rewarding, and fulfilling experiences associated with engaging in musical activities such as music listening [38]. However, enjoyment goes beyond the ability to merely perceive music and involves multiple dimensions spanning several fields of study. Neuroscience, psychology, and HL research have all investigated music enjoyment and provide unique perspectives on the topic.

This base of literature uses several terms interchangeably with music enjoyment, including music reward, music appreciation, and music appraisal [1,12,17,33,39]. When discussing individual studies, this review will use the term for music enjoyment used by the referenced studies themselves. Other common concepts in the music-enjoyment literature are music engagement and music perception. Music engagement is the degree to which someone interacts with music in their daily life. Engagement includes passive listening and active engagement with music such as singing or playing an instrument [40,41,42]. Music perception is the ability to identify elements of music such as pitch, melody, or rhythm [43].

### 3.1. Neuroscience and Music Enjoyment

Neuroscience research on music enjoyment emphasizes the role of music as a stimulus that can elicit pleasure and engage reward and motivation pathways [44,45]. There are several key components to the neuroscience of music enjoyment: dopamine-mediated response, the role of prediction and surprise, and interactions between the auditory and reward systems.

Studies have demonstrated a relationship between music reward and dopaminergic pathways, specifically the mesolimbic and mesocorticolimbic pathways. These pathways have been previously implicated in reward, emotion, motivation, and arousal [46]. Blood and Zatorre, 2001, performed positron emission tomography (PET) scans on participants who listened to a piece of music they had previously identified as enjoyable [46]. PET results revealed an increase in cerebral blood flow in areas of mesolimbic and mesocorticolimbic pathways that was associated with patient-reported sensations of “chills” [46]. These subjective, pleasurable sensations of “chills” and “shivers-down-the-spine” are often reported with pleasurable responses to music listening and have been used as a marker of peak emotional response to music [46,47,48].

Similarly, a 2011 PET and functional magnetic resonance imaging (fMRI) study of participants listening to music found that peak emotional response was associated with endogenous dopamine release from the striatum as well as activity in the nucleus accumbens [47]. To further characterize dopamine’s role in music enjoyment, Ferreri et al., 2019, performed a double-blind, crossover experiment [44]. This demonstrated that administration of a dopamine precursor (levodopa) increases participant-reported music hedonic (pleasure) and music motivation [44]. In this same trial, administration of a dopamine-antagonist (risperidone) produced the opposite effects, demonstrating a potential causal relationship between dopamine and musical reward [44].

The role of prediction, uncertainty, and surprise in musical pleasure has also been investigated [38,45]. An fMRI study by Gold et al., 2019, found that differences between expectations and experiences in music were correlated with activity in the nucleus accumbens, indicating that expectations have a role in enjoyment [45]. This prediction-based pleasure may be mediated by interactions of subcortical reward systems with cortical loops between auditory and frontal cortices [38].

Studies have highlighted this functional connection between the auditory cortical and subcortical reward structures. Salimpoor et al., 2013, found that the functional connectivity between the nucleus accumbens, auditory cortices, amygdala, and ventromedial prefrontal regions (as measured with fMRI) could be used to predict the reward value of a musical piece [49]. Martínez-Molina et al., 2016, investigated this theory of connectivity in patients with musical anhedonia: those who derive no pleasure from music despite normal perception and reward responses to other stimuli [50]. In these patients, there was reduced activity in the nucleus accumbens and decreased functional connectivity between the right auditory cortex and ventral striatum [50]. This functional connection between the right auditory cortex and striatum, as well as between the auditory cortex and amygdala, was redemonstrated by Mori and Zatorre, 2024 [51]. Together, these findings suggest that music enjoyment is related to a functional connection between the auditory cortex and reward structures.

### 3.2. Psychology and Music Enjoyment

Music psychology describes music enjoyment as a multifaceted concept influenced by interrelated factors [52]. Hedonic experience, motivation, emotional processing, musical identity, musical engagement, and the various functions that music serves are all considered contributors to music enjoyment [53,54,55,56,57,58,59,60]. Both neuroscience and music psychology have considered hedonic experience and motivation as aspects of music enjoyment [44,45,53]. However, music psychology approaches these concepts from a broader psychological rather than biological perspective.

Psychology divides motivation for engaging in musical activities into two sources: hedonic pleasure and eudaimonia [61]. Hedonia is defined as the act of consciously experiencing pleasure during music listening, and is identified as a source of motivation [53,61]. Hedonic motivation is then based in the desire to achieve pleasure or comfort, such as satisfying hunger or other basic bodily needs [62]. Eudaimonic motivation, however, is more nuanced. It is a positive feeling associated with a challenge or thought-provoking experience that leads to personal growth [54,55,61]. With respect to music, this could entail listening to a piece that sparks introspection or complex emotions [54]. Together, pleasurable, hedonic experience and eudaimonia may influence individuals to seek out and engage with music.

In addition to motivating music engagement, emotions are separately considered a key aspect of music enjoyment. Music has been shown to evoke a variety of emotions (e.g., awe and appreciation), and this emotional response is involved in musical enjoyment [56]. There is some discourse amongst the music psychology community about precisely how music evokes emotions. Several models of music emotional processing have been proposed, but recent theories have emphasized the importance of individual and contextual factors to emotional response [55,57,58].

Beyond pleasure and emotional responses, music enjoyment is influenced by personal relationships with music. Music identity is the integration of music into people’s view of themselves [56,59,60]. Individuals may identify as musicians, non-musicians, “hobby” or professional musicians, or have other perspectives about their relationship with music [59]. These self-perceptions may impact their enjoyment of and experience with music. Music engagement also impacts music enjoyment, and engagement can be impacted by musical training, preferences, or motivations [40,41,42].

Finally, music psychology has identified several enjoyable functions of music. Music has been shown to support mood regulation as well as social interaction and relatedness [55,60]. Music can be used to regulate stress, anger, anxiety, and loneliness, among other moods. Music can also foster communication, bonding, and a sense of belonging [55,60]. These social functions of music can be found across cultures and play an integral role in overall music enjoyment [63].

### 3.3. Hearing Loss Research and Music Enjoyment

While psychology has examined music enjoyment through this broad range of psychological and social dimensions, many HL studies have focused on perception as the marker of music enjoyment. Measures of music enjoyment in HL research have emphasized perceptual elements of music such as rhythm, pitch, timbre, and melody [25,26,30,39,64,65,66,67,68,69,70]. In addition, sound quality has commonly been used as a proxy for music enjoyment, with studies assessing naturalness, pleasantness, and musicality [12,16]. Other sound quality measures used in HL include reverberance, tinniness, and complexity [70,71]. In this perception-oriented conceptualization of HL and music enjoyment, music perception in HL patients is regarded as the sole factor influencing music enjoyment.

Although many HL studies have emphasized perception, broader conceptualizations of music enjoyment are also present in the field. Early work by Gfeller et al., 2000, created the Iowa Musical Background Questionnaire (IMBQ), a survey that assesses music appraisal separately from perception, along with music engagement and training [72]. Factors from music psychology such as music engagement, social value, and emotional response have been integrated into assessments of music enjoyment in HL [64,73,74]. Reviews by Riley et al., 2018, and Hwa et al., 2021, evaluated music assessment studies in CI users [25,26]. Riley et al., 2018, performed a systematic review of studies evaluating music enjoyment and perception in CI users [26]. Only studies that played musical excerpts were included, so survey studies alone were not assessed. This review found that 18/18 studies assessed music perception, but only 8/18 specifically assessed music enjoyment as well.

Hwa et al., 2021, investigated tools that assessed music perception or experience in CI users [25]. This included objective tools of music perception as well as subjective questionnaires. The content for eleven of these tools was described. Similar to Riley et al., 2018, a higher proportion of studies included perceptual components than enjoyment [26]. The majority (9/11) assessed at least one perceptual element (such as pitch discrimination, melody identification, timbre) [25,26]. Only 3/11 assessed subjective factors such as music experience, enjoyment, and/or quality of life, 2/11 included music engagement, and 3/11 music background [25]. While the HL field does investigate a broader approach to music enjoyment, much of the literature is perception-oriented.

## 4. The Relationship Between Hearing Loss and Music Enjoyment

The perceptual component of music enjoyment represents a unique challenge in hearing rehabilitation. Music is a particularly complex type of sound, characterized by multiple instruments and voices that dramatically vary in frequency, pitch, volume, and temporal presentation [27,28,29]. Speech, in comparison, has a more limited frequency range and requires comparatively less spectral resolution and temporal fine structure (TFS) processing [27,28,75]. In addition, pitch, timbre, and temporal processing of music are impacted by HL [27]. This makes music perception especially vulnerable to the impacts of HL.

HL can be categorized as sensorineural HL (SNHL), conductive HL (CHL), or mixed HL—a combination of SNHL and CHL [76]. SNHL, the most common form of HL, is characterized by damage to the cochlea and neural structures along the auditory pathway [76]. Damage to cochlear outer hair cells reduces the auditory system’s frequency selectivity, broadening auditory filters and making it difficult to distinguish between similar pitches [77]. Pitch perception can also be impacted by disruption of the cochlea’s tonotopic organization in HL. Normally, frequencies are mapped onto certain locations of the cochlea. However, in animal models of HL, distortion of this structure has been shown to leave auditory nerve fibers less responsive to their intended frequency [78,79]. This decreased frequency perception in HL has been associated with reduced recognition of melodies played with binaural pitch stimuli [80]. Collectively, these effects may make music listening more challenging for those with HL.

Auditory temporal processing refers to the brain’s ability to perceive, recognize, and differentiate acoustic signals and sound changes across precise timeframes. This is another important aspect of music perception that is degraded in HL and can be categorized into two components—temporal envelope processing and TFS. The temporal envelope is involved in slow amplitude fluctuations, while TFS processes rapid oscillations [81]. Temporal acuity is reliant on cochlear inner hair cells and the synapse ribbons which connect them to ganglion cells [82]. Cochlear synaptopathy, or “hidden” HL, which is characterized by subclinical changes, is of particular importance to temporal processing as it involves damage to synapse ribbons [83]. Speech perception primarily depends on temporal envelope processing, which remains generally intact in HL [81].

On the other hand, TFS processing is reliant on neural phase-locking (the ability of auditory nerve fibers to fire synchronously with individual cycles of a sound waveform), which depends on precise signal transmission at inner hair cell ribbon synapses and cochlear mechanical properties [84,85,86]. Though music perception also depends on envelope processing, it relies heavily on TFS, which contributes to music-relevant perceptual processes [84,87]. TFS is more vulnerable than temporal envelope processing to HL because it requires higher-precision neural coding and depends on narrow cochlear tuning that is degraded by outer hair cell loss [81,88,89]. Cochlear synaptopathy further degrades temporal coding [90]. As such, HL contributes to a degraded perception of pitch (i.e., ability to distinguish notes of two different frequencies), harmony (ability to identify and evaluate combinations of different musical pitches), sound segregation in complex auditory environment (e.g., understanding speech in an acoustically challenging environment such as a noisy restaurant or separating individual instruments or voices in polyphonic music), and timbre [84,88,91]. Timbre is the characteristic of a sound that allows a listener to distinguish between sounds [92]. For example, timbre is the aspect of sound that makes a flute and a violin sound distinct from one another, even if they are playing the same note at the same volume. This is a complex perceptual element that relies on spectrotemporal processing [93,94]. For patients with HL, perception of timbre may be impacted. Studies have shown a reduction in timbre differentiation for patients with steep HL and for CI users as a whole [27,95].

Since HL influences music perception differently than speech perception, hearing rehabilitation efforts geared toward speech do not necessarily lead to successful music listening. For example, modern hearing aids (HAs) are primarily designed for speech perception, so their signal-processing strategies are not optimized for music [29,96,97]. HAs have been shown to improve ratings of sound quality as compared to unaided listening, but many HA users continue to report challenges with music-listening [12,16,98]. HA compression of auditory input distorts the neural code for incoming speech and decreases spectrotemporal contrast [99]. This means that HAs do not fully rehabilitate frequency selectivity, which is a key component of music perception.

CI users may have even greater difficulty perceiving music than HA users [21,73,100,101]. Although the temporal envelope is preserved in CI users, spectral fine structure is compromised [102]. This means that rhythm is relatively preserved while spectrotemporal components such as pitch, timbre, and melody are markedly impaired [26,27]. This reduced spectrotemporal resolution is, at its core, due to a disconnect between device capabilities and biological processing norms [103]. CIs utilize an electrode array with a limited number of electrodes (~12–22) which cannot reproduce the spectral resolution provided by the tens of thousands of spiral ganglion neurons in the normal auditory system [102,103]. The limited number of stimulation channels, combined with current spread around adjacent electrode contacts, reduces spectral resolution due to “channel interaction,” resulting in overlapping interference between channels and smearing of the acoustic signal. Consequently, CI users experience a compressed representation of sound characterized by limited dynamic range and degraded spectrotemporal resolution.

Some HAs include music-listening programs that optimize parameters for music; however, they remain underutilized [97]. Effective listening programs and appropriate use of them may improve music perception. For example, it has been demonstrated that reducing the processing delay time of HAs can improve spectrotemporal performance [104]. CIs do not have an equivalent music program; however, some programming strategies have been investigated to improve music transmission [105]. Direct streaming is a more recent advancement that allows HAs or CIs to wirelessly connect to phones and other devices for audio streaming [106,107]. Streaming can produce a higher signal-to-noise ratio for speech than conventional listening by removing background noise [107]. Even with direct streaming, devices’ intrinsic processing constraints distort music and limit music perception.

## 5. When Perception and Enjoyment Diverge

Research indicates that music perception and enjoyment, while related, are not precisely correlated and should be considered interrelated but distinct constructs [1,33,34,35]. The literature on this topic emphasizes two themes. Worse hearing outcomes may be associated with decreased enjoyment, but personal and contextual factors can impact music enjoyment even when perceptual performance stays the same [1,15,17,73]. Practically, this can manifest in a number of ways. Patients may demonstrate preserved enjoyment despite more severe HL or reduced enjoyment even with preserved hearing, and individual variability exists along this spectrum.

In 2008, Gfeller et al. used a battery of music perception tests paired with a music appraisal questionnaire to investigate the predictors of perception and enjoyment in CI users [22]. Their multivariate analysis indicated that perception and enjoyment are separate outcomes predicted by different variables [22]. Other early work by Wright and Uchanksi, 2012, and Drennen et al., 2015, revealed similar findings: music perception and enjoyment are only weakly, if at all, related in CI users [31,32]. Looi et al., 2019, surveyed HA users and found that patients with severe HL had poorer appreciation scores than those with mild or moderate HL [15]. However, there was a great deal of variability within groups, suggesting that HL severity is not the sole predictor of music appreciation [15].

In 2016, a questionnaire paired with electrophysiological recordings from Bruns et al. investigated this relationship in a cohort of prelingually and postlingually deafened CI users [34]. This study compared music perception, music appreciation, and semantic processing [34]. A double dissociation was observed in which the onset of HL affected music perception and appreciation differently. Fuller et al., 2019, found similar results in CI users, with early-deafened, late-implanted CI users having greater music appreciation than postlingual users even though these groups had similar perceptual abilities [33]. Collectively, this work indicates that while perception and enjoyment are related, the underlying mechanisms are distinct.

Recent work from Gao et al., 2026, has shown that these findings persist when applied to a large cohort (*n* = 201) spanning the full spectrum of hearing [73]. This study included patients across the hearing spectrum, including individuals with normal hearing, unaided HL, HA users, and CI users [73]. As expected by the perception-oriented concept of music enjoyment, worsening hearing status was associated with decreased music perception [73]. However, there was no association between HL severity and overall reward, further supporting the theory that music perception and reward are distinct [73].

Subsequent work has identified several personal and environmental factors that may influence music enjoyment. A study by Gfeller et al., 2019, used qualitative methods to investigate what these factors may be. CI users were surveyed and interviewed to assess their music-listening experiences [17]. Their qualitative analysis demonstrated that CI users actively adapted their listening behaviors to improve their music enjoyment [17]. Reported strategies included managing expectations, modifying listening environment and social context, and using technological tools [17]. Specific adaptations included finding a quiet environment, using closed captioning, and selecting familiar music [17]. While these factors do not change a patient’s perceptual ability, they can improve enjoyment [17].

Other demographic factors and their impact on music enjoyment have been examined throughout the HL literature. Musical training has received particular attention, as musicians are thought to be disproportionately affected by degraded music perception due to their heightened sensitivity to changes in pitch, timbre, and sound quality [98,108,109]. However, studies have also demonstrated that music training and prior musical experience are associated with increased music enjoyment [1]. Indeed, one study found that musical training had a larger influence on music enjoyment than hearing status [1]. Past musical experience has also been associated with an emotional attachment to music itself, and these emotional responses are linked to music enjoyment [1]. Musical identity, particularly strongly identifying as a musician, may drive patients to invest more time and energy into their music experience and enjoyment [23]. Onset of HL and timing of hearing of CI implantation may also impact music enjoyment [33]. A study on CI users found that early-deafened, late-implanted CI users reported greater enjoyment than postlingually deafened controls [33]. Collectively, these findings suggest that personal characteristics, experiences, and motivations shape music enjoyment independently of auditory perception alone.

Focused listening practice or training has been investigated as a means to improve music enjoyment, but results are mixed. Findings suggest that specific aspects of music perception and enjoyment for CI users may be improved by formal training [24,110]. Several studies demonstrate that patients are interested in participating in some form of music training; however, training remains minimally available in clinical practice [17,24,111]. These studies suggest that intentional engagement with music may improve music enjoyment.

**Figure 1:**
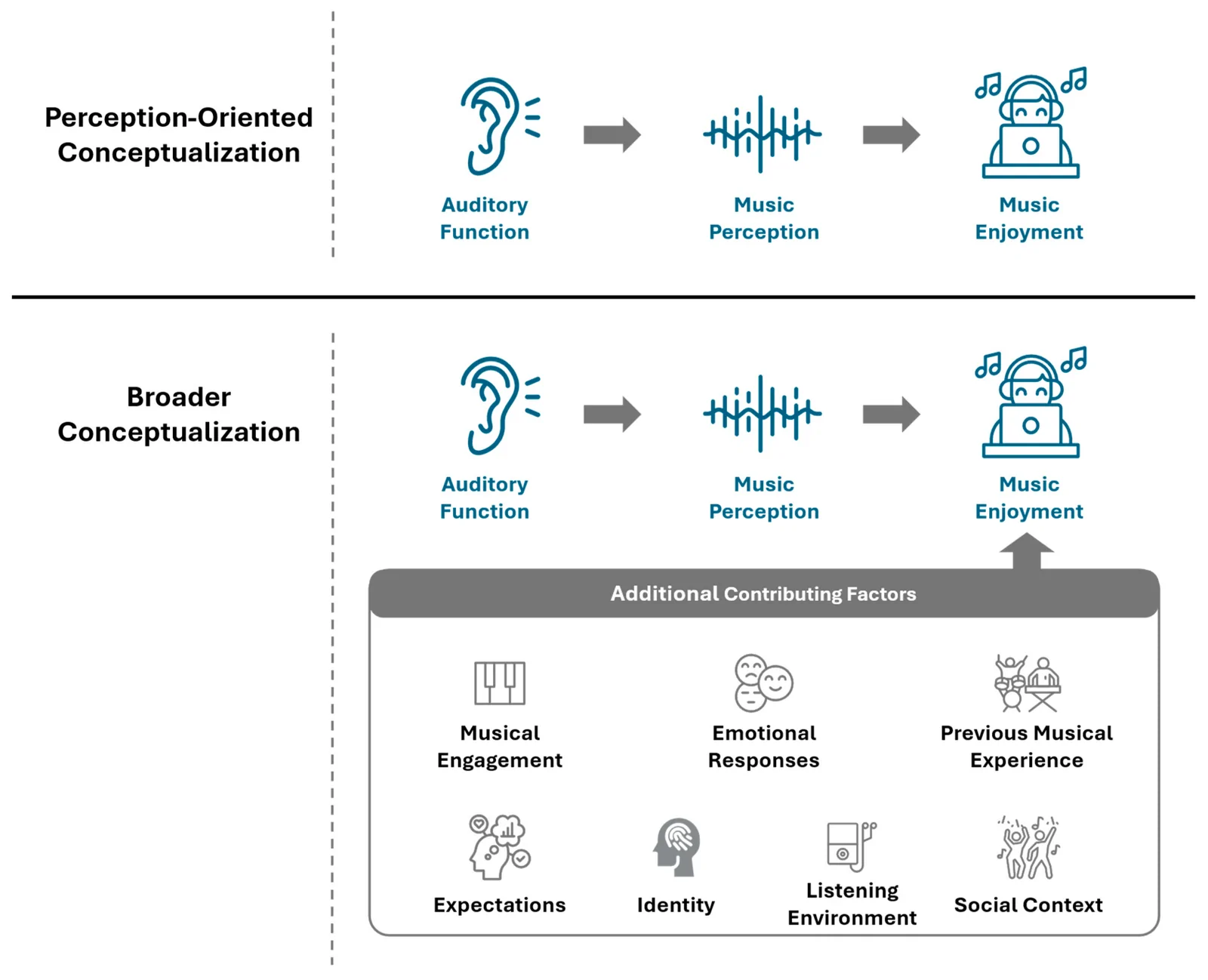
Conceptualizations of music enjoyment. The perception-oriented conceptualization is that HL impairs music perception, and reduced music perception directly results in reduced music enjoyment. This understanding implicitly conflates music perception with music enjoyment, despite these representing related but distinct constructs. The broader conceptualization recognizes that although auditory perception contributes to music enjoyment, there are several additional factors that further modify music enjoyment. This is based on an understanding that music perception and enjoyment are related but diverging constructs.

Collectively, this work supports a broader approach to music enjoyment in HL. Within this more comprehensive understanding, auditory perceptual changes are one factor influencing music enjoyment, alongside personal, behavioral, and contextual factors. The perception-oriented conceptualization is compared to the expanded conceptualization in Figure 1.

## 6. Lessons from Music Psychology and Neuroscience

As compared to HL science, psychology’s conceptualization of music enjoyment is more comprehensive and nuanced. Several of these psychology factors are now being incorporated into contemporary HL research, including emotional responses, musical identity, and social factors. As HL research into music enjoyment continues to evolve, music psychology and neuroscience may provide useful insights for conceptualization and measurement.

One area that may offer guidance is the use of patient-reported outcome measures (PROMs). PROMs are validated tools, often taking the form of questionnaires, that are used to formally collect patient-reported outcomes. PROMs can be used to assess a variety of patient reports, such as pain severity, quality of life, and anxiety symptoms [112,113]. A framework for the development of PROMs has been outlined by the National Institutes of Health Patient-Reported Outcome Measurement Information System (PROMIS) [114]. This includes an iterative process of development involving stakeholder engagement and psychometric validation of the instrument [115]. Please note, this abbreviation for PROMs is distinct from a similar abbreviation in the music perception field: PROMS, the Profile of Music Perception Skills [43].

Multiple well-established psychology-based PROMs assess aspects of music experience in the general population: the Goldsmiths Music Sophistication Index (Gold-MSI) and Barcelona Musical Reward Questionnaire (BMRQ). These are two widely used self-report instruments that assess different aspects of individuals’ relationships with music, including musical engagement, reward, emotional response, social reward, mood regulation, and connection to music [52,116]. The BMRQ is scored on the following factors: musical seeking, emotion evocation, mood regulation, social reward, and sensory motor [52]. It is formally validated to measure musical reward, a term which has been used synonymously with music enjoyment, appreciation, and music appraisal in the HL literature. The Gold-MSI scores active engagement, perceptual abilities, musical training, singing abilities, general musical sophistication, and emotions [116]. The Gold-MSI is broadly described as measuring musical sophistication and includes questions that explicitly address the music psychology aspects of music enjoyment outlined in Figure 1 [116]. Both PROMs were psychometrically validated in the nonclinical, adult population. Over the years, they have been adapted into other languages including German, Spanish, Portuguese, French, Chinese, Danish, Japanese, Turkish, and Italian [117,118,119,120,121,122,123,124,125].

The breadth of topics assessed by these instruments reflects the multidimensional nature of music enjoyment and the overall musical experience. Selected questions from the Gold-MSI and BMRQ illustrate how these PROMs evaluate the various aspects of music enjoyment that neuroscience and music psychology describe [52,116]. Questions are grouped below to demonstrate how the Gold-MSI and BMRQ address the music psychology concepts of motivation, emotional response, musical engagement, music identity, and social context, as well as the neuroscientific principle of “chills” or “shivers” being associated with peak pleasurable response. Some of these questions address multiple music psychology concepts but are only listed once for clarity.

Motivation:

*“I often pick certain music to motivate or excite me”*—Gold-MSI

*“Music can evoke my memories of past people and places”*—Gold-MSI

Emotional Response:

*“I am able to talk about the emotions that a piece of music evokes in me”*—Gold-MSI

*“I get emotional listening to certain pieces of music”*—BMRQ

*“Music calms and relaxes me”*—BMRQ

Musical Engagement:

*“I enjoy writing about music for example on blogs and forums”*—Gold-MSI

*“I have attended ___ the following number of live music events as an audience member in the past twelve months”*—Gold-MSI

Music Identity:

*“I would not consider myself a musician”*—Gold-MSI

*“I have never been complimented for my talents as a musical performer”*—Gold-MSI

Social Context:

*“When I share music with someone I feel a special connection with that person”*—BMRQ

*“Music makes me bond with other people”*—BMRQ

Chills or Shivers:

*“I sometimes choose music that can trigger shivers down my spine”*—Gold-MSI

*“I sometimes feel chills when I hear a melody that I like”*—BMRQ

Neuroscience research has investigated the connections between Gold-MSI and BMRQ scores and neurologic findings. Loui et al., 2017 found that the volume of tracts connecting cortical and reward structures can predict musical reward, as measured by the BMRQ [126]. A resting-state fMRI study by Cui et al., 2023, found a possible association between Gold-MSI subscores and sensory- and motor-area connectivity [127]. Another study by Cui et al., 2024, found linear relationships between arcuate fasciculus volume and aspects of the Gold-MSI [128]. Collectively, this work from music psychology and neuroscience suggests that the domains assessed by the Gold-MSI and BMRQ are associated with neurologic findings.

Although the Gold-MSI and BMRQ were not specifically designed for use in the HL population, they have occasionally been administered to HL patients [34,73,129]. Importantly, the need for a measure of music enjoyment that is valid across the spectrum of hearing does not depend on the assumption that hearing loss uniformly reduces overall music enjoyment. Rather, as hearing loss worsens, individuals may experience different trajectories across distinct domains of music enjoyment. For example, enjoyment related to sound quality or perceptual clarity may decline, while emotional connection, social engagement, nostalgia, or other rewarding aspects of music may remain relatively preserved [130]. Individuals may also adapt their listening behaviors, environments, or hearing device use to maintain enjoyment despite increasing auditory limitations. Thus, similar levels of global music enjoyment may mask substantial heterogeneity in how hearing loss affects specific dimensions of the musical experience. The Gold-MSI and BMRQ were designed to assess musical sophistication and reward, respectively, in the general population and provide important frameworks for understanding these constructs. However, neither was developed through stakeholder engagement across the spectrum of hearing or specifically designed to capture how hearing ability and hearing-related adaptations may shape these experiences. PROMIS guidelines emphasize stakeholder involvement and psychometric validation within the population and context of intended use [115]. Thus, a measure developed and validated across the hearing spectrum could complement existing instruments by capturing hearing-related dimensions of music enjoyment while retaining the broader insights established by music psychology.

The only music experience PROM validated for use in the HL population is the Music-Related Quality of Life (MuRQoL) [30,64]. This PROM was developed in 2017 by Dritsakis et al. for use in CI users. It assesses various aspects of music experience and music-related quality of life, including music enjoyment [30,64]. The MuRQoL has since been adapted into several other languages, including Italian, Spanish, and Turkish [131,132,133]. The MuRQoL consists of 36 5-point Likert-scale questions that assess music-listening abilities, attitudes, engagement, and related quality of life. It includes questions that are targeted to the HL population and specifically asks respondents about their enjoyment of music. One such question asks, “how important is it for you to enjoy music in noisy environments when no visual cues are available [30]?” Questions such as this assess music enjoyment in a manner that is appropriate to those with HL.

While the MuRQoL represents a meaningful step toward better assessment and conceptualization of music enjoyment in the HL population, its specificity to CI users limits its scope. Music perception and enjoyment represent a challenge for individuals across the full spectrum of HL [73]. Currently, no music-enjoyment PROM exists that is applicable to the general HL population.

## 7. Future Directions

This multidisciplinary synthesis of the music enjoyment literature identifies several opportunities for future research and clinical practice. First, the conceptual definition of music enjoyment as a multidimensional construct needs to be further developed and clarified. As proposed in this review, integrating perspectives from hearing science, music psychology, and neuroscience may provide a more robust conceptual understanding. Additional research is needed to further characterize how domains such as emotional responses, musical engagement, prior musical experiences, expectations, music identity, social context, and listening behavior contribute to enjoyment. A stronger conceptual definition would improve understanding of existing research and provide a foundation for future studies, hearing rehabilitation strategies, and patient-reported outcome measurement.

Clinically, future hearing rehabilitation interventions may benefit from incorporating music-specific counseling, listening strategies, and structured music training for interested patients. Qualitative studies have demonstrated that many individuals already employ adaptive strategies to improve their music-listening experience. Integrating the discussion of these approaches into hearing rehabilitation may help improve music-related outcomes. Continued refinement of music-listening programs for HAs and optimization of CI programming strategies may further improve music-related outcomes.

Finally, there remains a need for a validated music-enjoyment patient-reported outcome measurement across the hearing spectrum. Although the creation of the MuRQoL for CI users was an important advancement, no music-enjoyment PROM currently exists for the broader HL population. Future instrument development should incorporate stakeholder engagement and contemporary PROM development guidelines. A music enjoyment PROM would assess multiple dimensions of the music experience and incorporate lessons from music psychology. This would facilitate patient-centered research and provide a standardized method for evaluating music-enjoyment outcomes in HL patients.

## 8. Conclusions

Music listening is a meaningful activity that impacts quality of life for individuals with HL. While some HL research emphasizes music perception, existing evidence indicates that perception and enjoyment are related but distinct outcomes. Auditory perception contributes to music enjoyment but is not the sole factor defining it. Work from neuroscience, music psychology, and HL research supports a broader understanding of music enjoyment as a multidimensional patient-centered outcome incorporating perceptual, affective, behavioral, and contextual influences. Research suggests that music enjoyment is shaped by emotional responses, musical engagement, prior musical experiences, expectations, music identity, social context, and listening behaviors.

This broader conceptualization has important implications for outcome measurement. To develop and validate a PROM capable of assessing music enjoyment across the hearing spectrum, a clear conceptualization of music enjoyment is needed. The development of this PROM would support evaluation and rehabilitation for patients across the spectrum of HL.

## Data Availability

No new data were created or analyzed in this study. Data sharing is not applicable to this article.

## Abbreviations

The following abbreviations are used in this manuscript:

BMRQ	Barcelona Music Reward Questionnaire
CHL	Conductive Hearing Loss
CI	Cochlear Implant
HA	Hearing Aid
HL	Hearing Loss
fMRI	Functional Magnetic Resonance Imaging
Gold-MSI	Goldsmiths Music Sophistication Index
IMBQ	Iowa Musical Background Questionnaire
MuRQoL	Music-Related Quality of Life
PET	Positron Emission Tomography
PROM	Patient-Reported Outcome Measure
PROMIS	Patient-Reported Outcome Measurement Information System
PROMS	Profile of Music Perception Skill
SNHL	Sensorineural Hearing Loss
TFS	Temporal Fine Structure

## Appendix A

Searches were conducted on PubMed, Scopus, and Cochrane using combinations of defined search terms and searching English-language publications. Searches were conducted from database inception through 20 July 2026. Additional references were identified through manual screening of reference lists from included articles. The number of results returned by each database is presented below in Table A1.

PubMed was searched within all fields. Scopus was searched within article title, abstract, and keywords. Cochrane was searched within all text and allowed search word variations.

**Table A1:** Search Queries and Number of Results.

Search Query	PubMed (n)	Scopus (n)	Cochrane (n)
(“music enjoyment” OR “music reward” OR “music appreciation” OR “music appraisal”) AND “music psychology”	54	4	1
(“music enjoyment” OR “music reward” OR “music appreciation” OR “music appraisal”) AND “neuroscience”	37	7	0
“music perception” AND “hearing loss”	135	4	18
“pitch” AND “hearing loss” AND “music perception”	68	86	1
“timbre” AND “hearing loss” AND “music perception”	34	50	3
“temporal processing” AND “music perception”	14	22	1
“music perception” AND “hearing aid”	49	76	5
“music perception” AND “cochlear implant”	278	387	25
“music perception” AND (“music enjoyment” OR “music reward” OR “music appreciation” OR “music appraisal”)	66	83	5
“music perception” AND (“music enjoyment” OR “music reward” OR “music appreciation” OR “music appraisal”) AND “hearing loss”	18	26	1
(“music enjoyment” OR “music reward” OR “music appreciation” OR “music appraisal”) AND “music psychology”	54	4	1
(“music enjoyment” OR “music reward” OR “music appreciation” OR “music appraisal”) AND “neuroscience”	37	7	0
“music perception” AND “hearing loss”	135	4	18
“pitch” AND “hearing loss” AND “music perception”	68	86	1
“timbre” AND “hearing loss” AND “music perception”	34	50	3
“temporal processing” AND “music perception”	14	22	1
“music perception” AND “hearing aid”	49	76	5
